# Hand-Book for Hospitals

**Published:** 1896-07

**Authors:** 


HAND-BOOK FOR HOSPITALS. By ABBY HOWLAND WOOLSEY, Member of the Committee on Hospitals, State Charities Aid Association. No. 32 New York State Charities Aid Association Series. Third edition. Price, $1.00. New York: G. P. Putnam's Sons, 27 West Twenty-third street. 1895.

The revision of this useful manual was placed in the hands of Drs. Hitchcock, Wheelock and Wylie, who have carefully done their work. The opening chapter is useful alike to large and small hospitals. The paragraphs on art of planning are especially valuable. The hospital buildings are considered in chapter II., the air supply, heating, drainage and water supply in chapters III. and IV. In chapters VII. and VIII. the matron and head nurse can find hints which must be of service to them, and it is to be hoped that all nurses who read the section on coöperation in hospital work will profit thereby. Chapter XII., on disinfection, is thorough, but not up to date. The appendix gives the rules and general laws governing some of our largest and best conducted hospitals. The volume commends itself to all interested in hospital work. L. C. R.

